# Degree of Freedom of Gene Expression in Saccharomyces cerevisiae

**DOI:** 10.1128/spectrum.00838-21

**Published:** 2022-03-01

**Authors:** Zhen Yang, Feng Xu, Aijuan Xue, Hong Lv, Yungang He

**Affiliations:** a Shanghai Fifth People’s Hospital, Fudan University, Shanghai, China; b Shanghai Key Laboratory of Medical Epigenetics, International Co-Laboratory of Medical Epigenetics and Metabolism (Ministry of Science and Technology), Institutes of Biomedical Sciences, Fudan University, Shanghai, China; c State Key Laboratory of Genetic Engineering, School of Life Science, Fudan University, Shanghai, China; d Shanghai Engineering Research Center of Industrial Microorganisms, Shanghai, China; University of Mississippi

**Keywords:** *Saccharomyces cerevisiae*, coordinate system, degree of freedom, gene activity, gene expression

## Abstract

The complexity of genome-wide gene expression has not yet been adequately addressed due to a lack of comprehensive statistical analyses. In the present study, we introduce degree of freedom (DOF) as a summary statistic for evaluating gene expression complexity. Because DOF can be interpreted by a state-space representation, application of the DOF is highly useful for understanding gene activities. We used over 11,000 gene expression data sets to reveal that the DOF of gene expression in Saccharomyces cerevisiae is not greater than 450. We further demonstrated that various degrees of freedom of gene expression can be interpreted by different sequence motifs within promoter regions and Gene Ontology (GO) terms. The well-known TATA box is the most significant one among the identified motifs, while the GO term “ribosome genesis” is an associated biological process. On the basis of transcriptional freedom, our findings suggest that the regulation of gene expression can be modeled using only a few state variables.

**IMPORTANCE** Yeast works like a well-organized factory. Each of its components works in its own way, while affecting the activities of others. The order of all activities is largely governed by the regulation of gene expression. In recent decades, biologists have recognized many regulations for yeast genes. However, it is not known how closely the regulation links each gene together to make all components of the cell work as a whole. In other words, biologists are very interested in how many independent control factors are needed to operate an artificial “cell” that works the same as a real one. In this work, we suggested that only 450 control factors were sufficient to represent the regulation of all 5800 yeast genes.

## INTRODUCTION

Inspired by Feynman’s famous lecture in 1959, titled “There’s plenty of room at the bottom,” many scientists have considered life on the basis of a mechanical philosophy in which the entire cell is viewed as a factory containing elaborate protein machines with ordered movements (1). Remarkable advances in molecular biology over the past few decades have revealed that cell functions are carried out by macromolecular complexes containing multiple units with specific roles (2). Starting from molecular complexes or subsystems, biologists have provided new insights into cellular function. For example, in the early era of molecular biology, studies of the *lac* operon from Escherichia coli clarified the organization, regulation, and engineering of biological subsystems (3). The rapidly growing field of structural biology has supported the trend of conceptualizing proteins and other macromolecular complexes as molecular machines (4). Ambitious efforts have been made to understand and simulate cell functions as a collection of mechanical components (5–8).

Dependency is a basic rule of life. In his book “*What is Life?*,” Erwin Schrödinger suggests that life feeds on negative entropy, or free energy, to maintain dependency and protect against thermodynamic damage (9). It is therefore important to consider the dependency and regulation between and within different biological components. The primary goal of systems biology is to quantitatively model the interdependency among components of complex biological systems using a holistic approach (10, 11). In state-space representation, gene expression levels can be interpreted as outputs of a system and determined by several state variables of the system (12). The degree of freedom (DOF) is the minimum number of state variables that can thoroughly represent the state of a system. Thus, clarifying the DOF of gene regulation and how it is affected by biological factors is an important step toward developing a holistic understanding of a biological system (13).

Within a single cell, the interdependency of genetic regulation is linked with gene expression. The interdependency effectively reduces the systematic DOF of gene expression (14). On the other hand, while regulation redundancy is common in biological pathways, redundancy increases the DOF of gene expression by weakening the link between genetic regulation and gene expression (15, 16). Changes in gene transcription are the interplay between the regulation interdependency and pathway redundancy. To understand the interplay, changes in transcriptional states must be clearly represented by state variables (17, 18). A coordinate system of state variables allows a huge amount of transcriptional data to be represented and analyzed easily, even when the data are collected from different studies.

Saccharomyces cerevisiae, with ∼5,800 genes, is a useful model for exploring biological processes and molecular mechanisms (19). In the present study, we investigated the DOF of gene expression in S. cerevisiae to analyze transcriptional regulation using more than 11,000 data sets. Our findings indicated that the DOF of gene expression is astonishingly limited and much smaller than the total number of genes. The limited DOF indicates that gene expression in S. cerevisiae can be effectively represented by no more than 450 specific state variables in a state-space representation. Further analysis suggested that the representative variables can be interpreted by sequence motifs within promoter regions and gene ontology (GO) terms. Because the state variables serve as the bases for transcriptional states in linear space, we further developed a universal coordinate system to map the transcription states of S. cerevisiae and compare them among different replications and experiments.

## RESULTS

### DOF of gene expression in S. cerevisiae.

**FIG 1:**
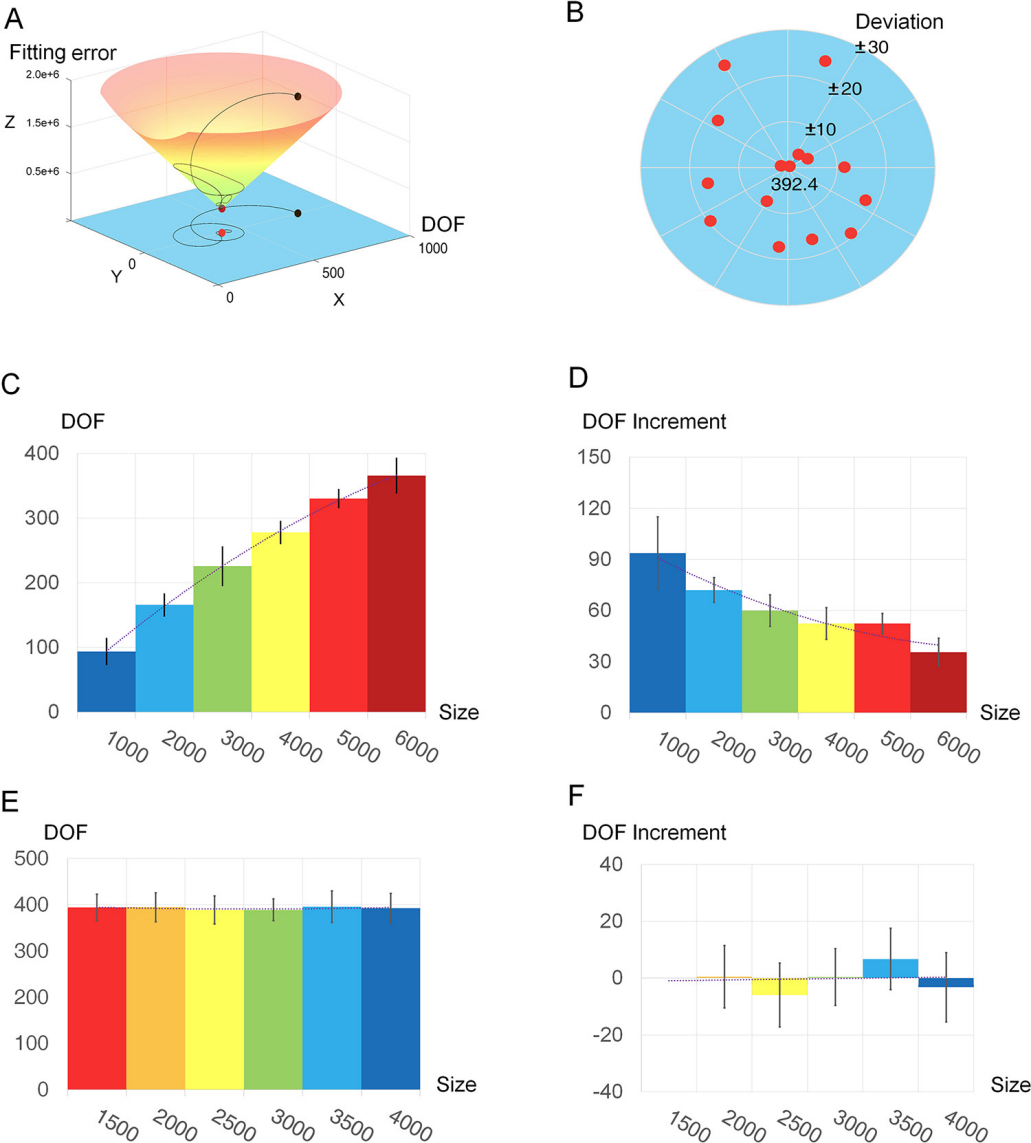
Determining the DOF of gene expression. (A) A diagram of the golden section search for determining the DOF. The search trajectory is presented by the black curve and projected as a modified Fibonacci spiral on an *x*-*y* plan (in blue), where *x* (DOF) is the input of loss function and *y* is shown as an imaginar*y* axis for convenience of visualization. When the output of loss function (fitting error) is given on the *z* axis, we present the whole search procedure in a three-dimensional (3-D) diagram. This search starts with an initial value of 800 (black ball) and reaches a final value of 390 (red ball). (B) This figure presents all 15 results in a scatterplot with polar coordinates. The radial coordinates indicate deviations of estimations to their average, and the angular coordinates are given to the 15 scatter points by evenly partitioning the 2π radians in order of estimation runs. (C) The number of estimated DOFs increases slowly with an increase in the amount of experimental data. The *x* axis shows the size of experimental data. (D) The increment of the estimated DOFs decreases with an increase in the amount of experimental data. The size of experimental data is presented in the *x* axis. (E) The number of involved genes has little effect on the DOF estimation. (F) The increase in DOF is approximately zero with different numbers of genes.

We determined the minimum number of state variables in the state-space model of genetic regulation for yeast genes (see Materials and Methods for details), i.e., the DOF of yeast gene expression, by applying a search strategy with cross-validation (20). Our study included 11,483 gene expression data sets and a total of 6,692 genes. After quality control procedures, 6,322 data sets with 4,529 genes were used in the investigation of cross-validation. In each cross-validation, the full data set with 4,529 filtered genes was randomly divided into a training set with 4,000 genes and a testing set with the remaining 529 genes. During the iterations, the search range for the DOF was continuously narrowed until the fitting error of the state-space model reached a minimum (Fig. 1A). DOFs of gene expression in the 15 cross-validations ranged from 368 to 418, with a mean of 392.4 and a standard deviation of 16.29 (Fig. 1B). These findings indicate that the DOF of gene expression in S. cerevisiae is much smaller than the total number of genes.

To investigate the robustness of the above-described analysis, we further estimated the DOFs in different scenarios with multiple empirical data sets generated by resampling. Using different data sets comprising 1,000 to 6,000 experiments, the estimated DOF became larger as the number of experiments increased (Fig. 1C). When including only 1,000 experiments, the estimated DOFs from 15 different evaluations averaged 93.53 with a standard deviation of 8.49. When 6,000 experiments were included, the mean estimated DOFs increased to 365.75 with a standard deviation of 8.49. The increments in the estimated DOFs were significantly reduced as the number of experiments increased (Fig. 1D). Comparison between 1,000 and 2,000 experiments revealed an increase of 72.07 in the mean estimated DOFs. When using the mean estimated DOFs of 5,000 and 6,000 experiments, the increase was reduced to 35.53.

We then fitted a quadric curve to the above-mentioned estimations to investigate the possible DOFs of gene expression over more experimental data. This type of extrapolation is very helpful toward further exploring the impact of data size. The fitted curve approximated a maximum of approximately 443 for a data set of about 11,000 experiments. This result was supported by the reduced increases in the estimated DOFs as the number of experiments in the data set increased. A fitted linear model suggested that the increasing amplitude would approximate zero as the data set increased to 10,000 experiments or more. Surprisingly, changing the number of genes had little effect on estimation of the DOF. In evaluations using all the experimental data but with different numbers of genes (ranging from 1,500 to 4,000), the mean estimated DOF was approximately 390 (Fig. 1E). The mean DOF did not differ significantly among estimations with different numbers of genes when analysis of each gene size was applied to 15 randomly generated data sets (Fig. 1F). This finding suggested that a small group of genes is capable of recapitulating the global dynamics of the state variables of gene expression.

### Biological features interpreting the state variables of transcriptional regulation.

**FIG 2:**
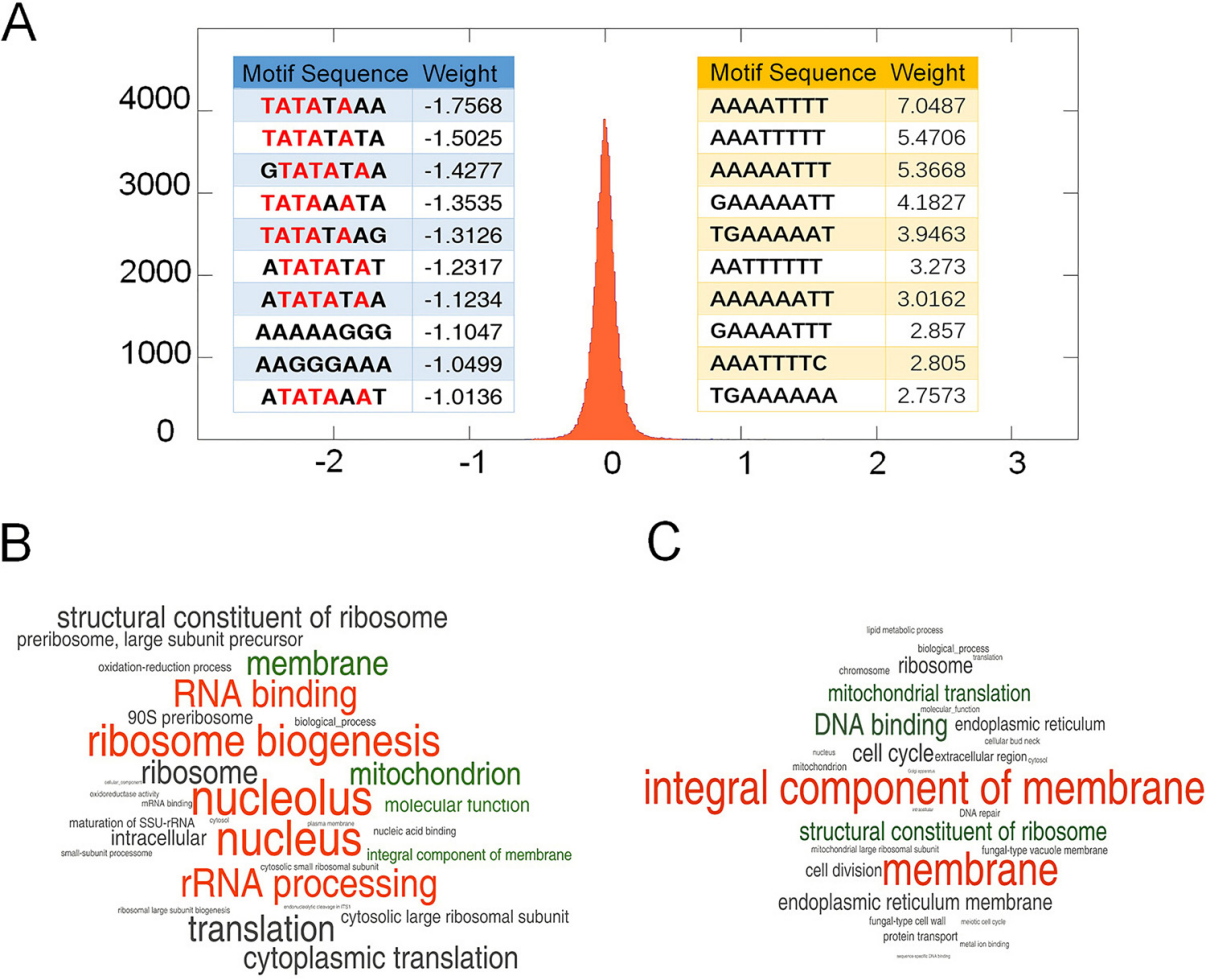
Sequence motifs and ontology terms explain the state variables. (A) Sequence motifs with extreme contributions to the primary state variable. Bell curve showing the distribution of weights for all the sequence motifs. Weight is provided on the *x* axis, and density is presented on the *y* axis. The tables present the most significant sequence motifs, and the typical TATA boxes are highlighted in red. (B and C) Two word clouds of GO terms interpret the 1st (B) and 19th (C) state variables. The GO terms with significant positive weights are highlighted in orange, while the terms with significant negative weights are highlighted in green.

To interpret the state variables corresponding to different DOFs in the state-space representation of gene expression, we searched 8-mer sequence motifs within the 500-bp regions upstream from each transcription start site in the yeast genome. The weights (contributions) of each sequence motif on different state variables were obtained by fitting the present motifs of each gene to the variables using the least-squares method (see Materials and Methods). Our results revealed that different sequence motifs made significantly different contributions to the various state variables. For the primary variable, for example, 62 motifs had weights of less than −0.5 and 179 motifs had weights larger than 0.5, while the total mean weight was approximately 0 (−7.199 × 10^−4^) with a standard deviation of 0.121. Twenty motifs with the most extreme weights are shown in Fig. 2A. The most well-known TATA box was found in 8 of 10 motifs with extreme negative weights. Approximately 20% of the yeast genes contained the TATA box with the consensus sequence 5′-TATA(A/T)A(A/T)-3′ (21). The top 10 motifs with extreme positive weights had a low sequence complexity with multiple A’s and T’s (Fig. 2A). This finding suggests that A/T-rich motifs play important roles in regulating gene transcription.

Gene ontology can also be used to interpret differences among state variables by fitting GO terms to the variables (see Materials and Methods). For example, in Fig. 2B and C, we present GO terms with significant contributions (weights) to the 1st and 19th state variables (see the last paragraph of Results for details about further work regarding data visualization of the 19th state variable). GO terms related to protein production, such as “nucleolus,” “ribosome biogenesis,” “RNA binding,” and “rRNA processing,” made significantly positive contributions to the 1st state variable. On the other hand, “membrane,” “mitochondrion,” and “integral component of membrane” made significantly negative contributions to the 1st state variable. Surprisingly, “mitochondrion” was a negative contributor for the 19th state variable as well, but the other terms, “integral component of membrane” and “membrane,” were the top positive factors (Fig. 2C). Other terms, such as “structural constituent of ribosome” and “nucleus,” also had different effects on the 1st and 19th state variables. Our findings suggest that the state variables are interpretable with GO terms and other integrated biological information.

### Information capacity of motifs restricting the gene expression complexity.

**FIG 3:**
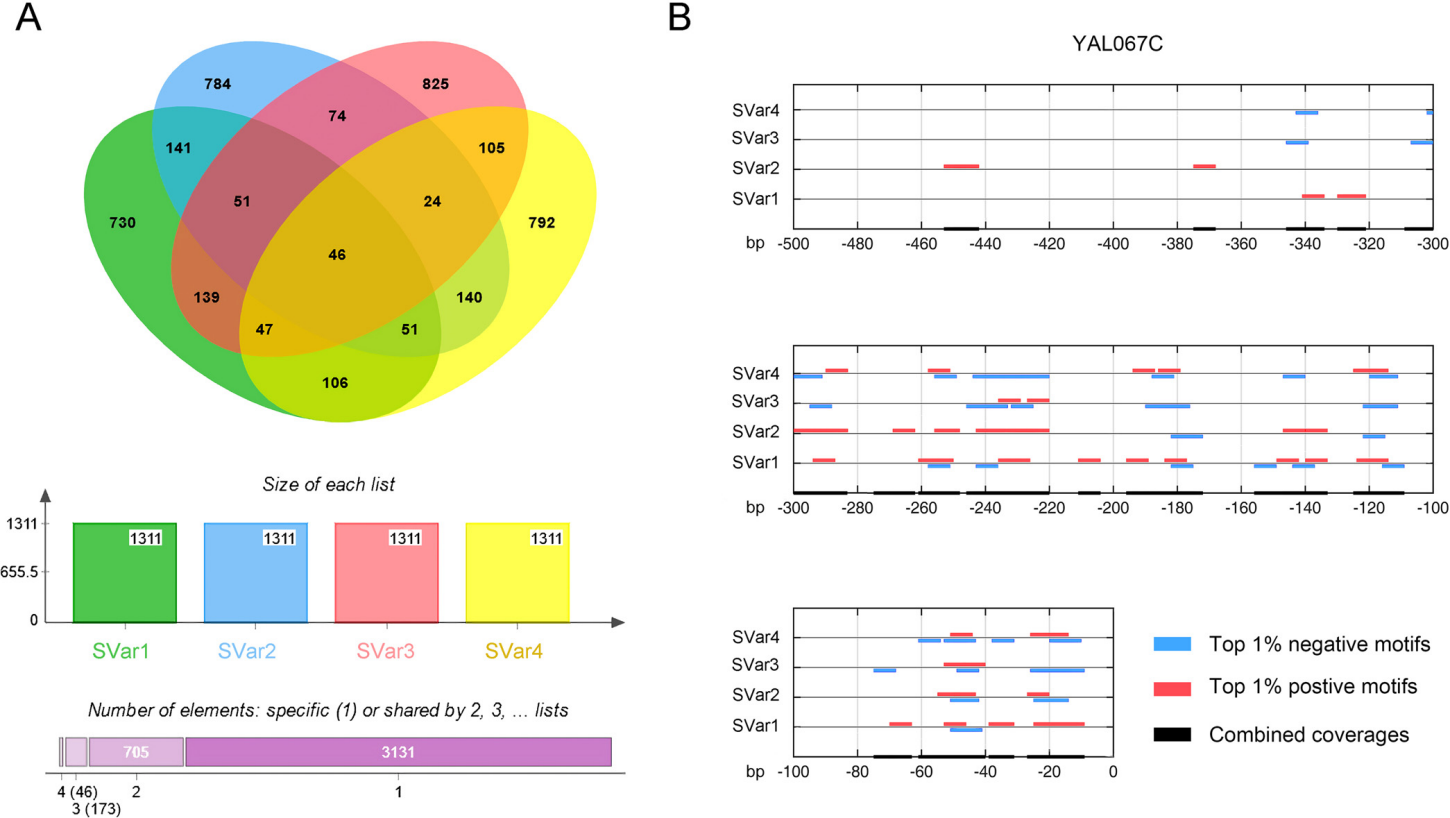
Motif sharing among the 1st to 4th state variables (SVar). (A) The sharing of critical motifs among the 1st to 4th state variables. (B) This figure presents the map of critical motifs in the promoter region of gene *YAL067C*.

The limited size of state variables in gene expression suggests that the regulation network may not be capable of simultaneously transmitting a great amount of regulatory information. We therefore evaluated the information capacity of motifs critical for regulation of the gene expression network. The information capacity is a good indicator for the binding specificity of protein factors in the presence of diversifying sequence motifs. We selected a set of 1,311 motifs for each state variable in which each of the motifs was ranked in the top 1% of positive- or negative-weight motifs of the corresponding state variable (see Materials and Methods for details). Our analysis revealed that the selected motifs of a total of 450 state variables had a Shannon information capacity of 5.46 bits. The information capacity was much smaller than the theoretical maximum (16 bits for 8-mer motifs). Shannon’s capacity theorem suggested that a limited information capacity makes it difficult to transmit abundant regulatory signals without a loss of information (22). To further explore the selected motifs, we investigated the overlaps of the 450 motif sets of different state variables. The overlaps of motif sets for the 1st through 4th state variables are presented as an example in Fig. 3A. Our analysis showed that nearly half of the selected motifs in each set were shared with another motif set. Although the overlaps were significant, the majority of the motifs (3,131 motifs in total) appeared in only 1 of the 4 sets. Besides 705 motifs in at least 2 sets and 173 motifs in 3 sets, 46 selected motifs were found in all 4 motif sets. To investigate the sharing of selected motifs over different state variables, we mapped the selected motifs to the promoters of different genes (the 500-bp region upstream from each transcription start site). The results demonstrated that the majority of selected motifs in each promoter were shared by different state variables (see Fig. 3B for an example). Our analysis of motif sharing supports the notion that the information capacity of sequence motifs in promoter regions is limited due to the sharing among state variables.

**FIG 4:**
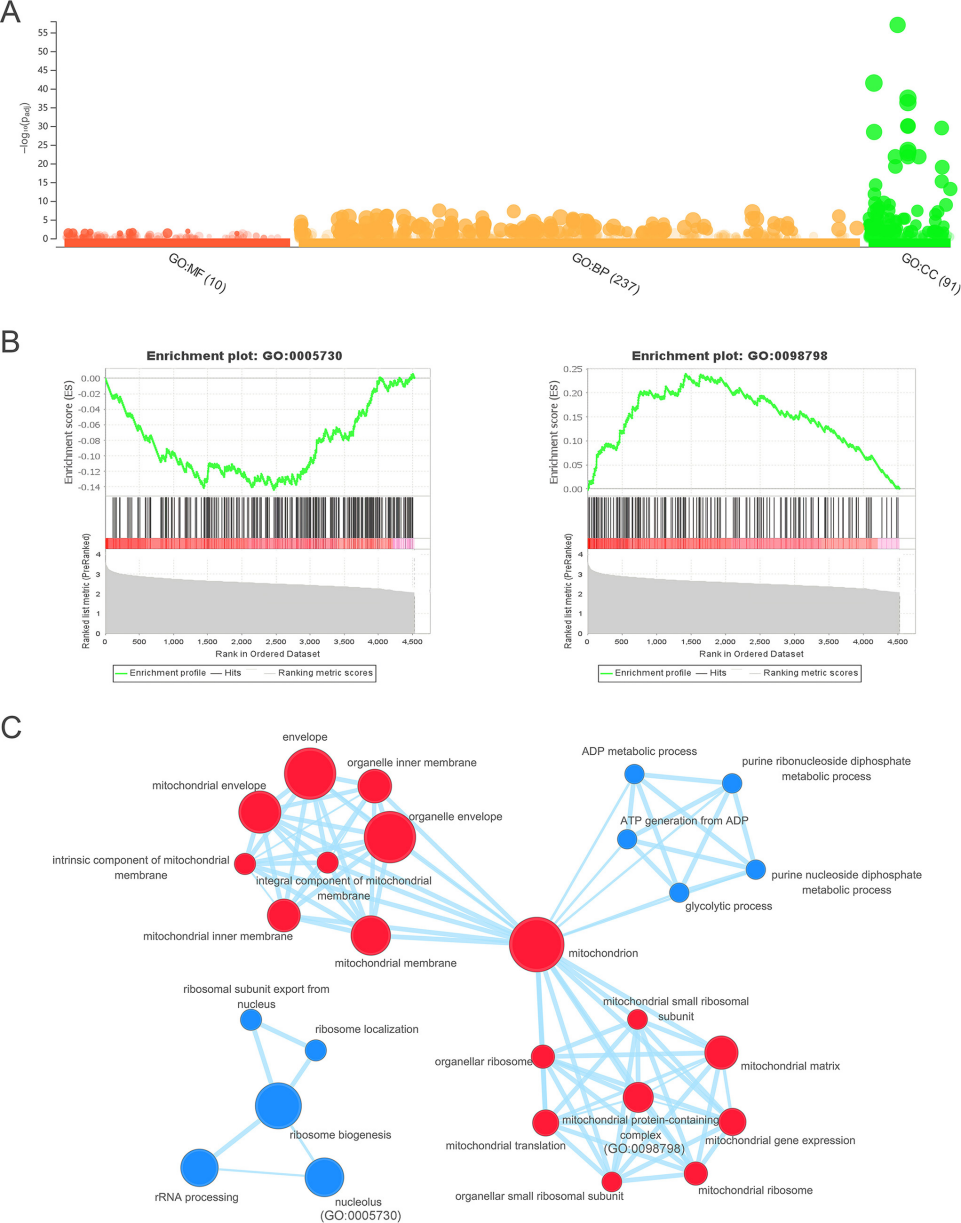
Significant enrichments of ranked genes in different GO terms. (A) The Manhattan plot from g:Profiler shows statistical significance of enrichment analysis for GO terms. The numbers in parentheses present the sizes of significant enrichments in each GO category. (B) The figures from GSEA show the results of the enrichment analysis for GO:0005730 (nucleolar components) and GO:0098798 (mitochondrial protein-containing complex). (C) An enrichment map of some significant GO terms. Gene promoters of mitochondrial components (nodes in dark red) have higher information capacity than the others, while those of the nucleolus, ribosomes, and metabolic processes (nodes in steel blue) have low information capacity. The areas of the nodes are proportionate to the numbers of genes in the GO terms, and the links between nodes indicate similarity (≥50%) of the gene contents between corresponding GO terms.

To better understand the motifs, we calculated Shannon’s information capacity for the presence of critical motifs of each promoter (see Materials and Methods for details). Our results revealed that the information capacity varies according to their presence in different promoters, with an average of 2.53 bits and a standard deviation of 0.26 bits. We conducted an enrichment analysis of GO terms on the gene list that was ranked based on the information capacities of the selected motifs in the gene promoters. The analysis detected the clustering tendency of member genes of each GO term in the ranked gene list (23, 24). Our results revealed significant enrichments in a total of 334 GO terms of molecular function, cell components, or biological processes (Fig. 4A). For example, 285 promoters for genes that encode components of the nucleolus (GO:0005730) had significantly low information capacities among promoters of the total of 4,529 genes, with the normalized enrichment score being −3.29 (false discovery rate [FDR] *q* value of ≤0.001) (Fig. 4B, left). In contrast, 170 gene promoters of the mitochondrial protein-containing complex (GO:0098798) had a much larger information capacity than the other gene promoters, with a normalized enrichment score of 2.81 (FDR *q* value of ≤0.009) (Fig. 4B, right). Besides the GO term of mitochondrial protein-containing complex, multiple other GO terms related to mitochondrial components with highly informative promoters were identified (Fig. 4C). Our study indicates that gene promoters have different information capacities for the presence of critical motifs and that the differences are associated with their biological characteristics.

### Universal coordinate system presenting the state of transcription.

**FIG 5:**
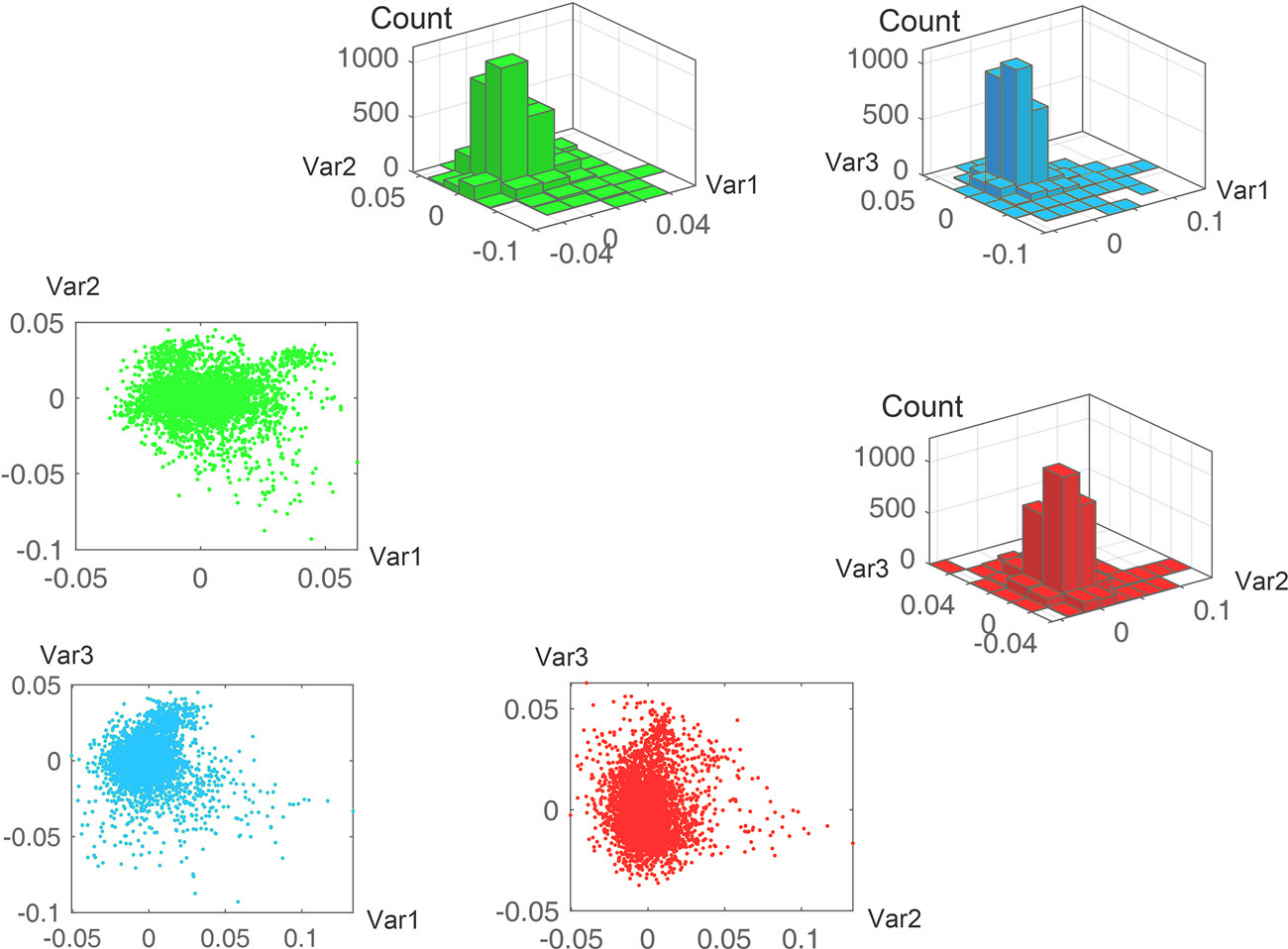
The state variables of the transcriptional model contributed independently to the expression of different genes. The scatterplots show the load of each gene in the 3 state variables, and the 3-D histograms show the densities of the genes. The 1st, 2nd, and 3rd state variables explain 25.1%, 6.4%, and 3.4% of the total variance, respectively.

The state variables in the gene expression model provide the opportunity to develop a coordinate system for presenting the state of transcription (25). Because the analysis suggested that the DOF of gene transcription was approximately 450 in yeast, we established a universal coordinate system designated transcriptional coordinate system of yeast (TCSY) with 450 dimensions. Each dimension presents a single state variable. The coordinate system enables us to map any transcriptional state of cells to the high-dimensional space of TCSY (see Materials and Methods). Our study of 6,322 experiments suggests that TCSY explains 89.3% of the total variance and the 1st, 2nd, and 3rd state variables explain 25.1%, 6.4%, and 3.4% of the total variance, respectively. The different variables made various contributions to the expression of different genes (Fig. 5). With the rapid increase in the accumulating variance explained by the top variables, the variance explained by a single variable drops below 1.0% for the 11th variable and thereafter.

**FIG 6:**
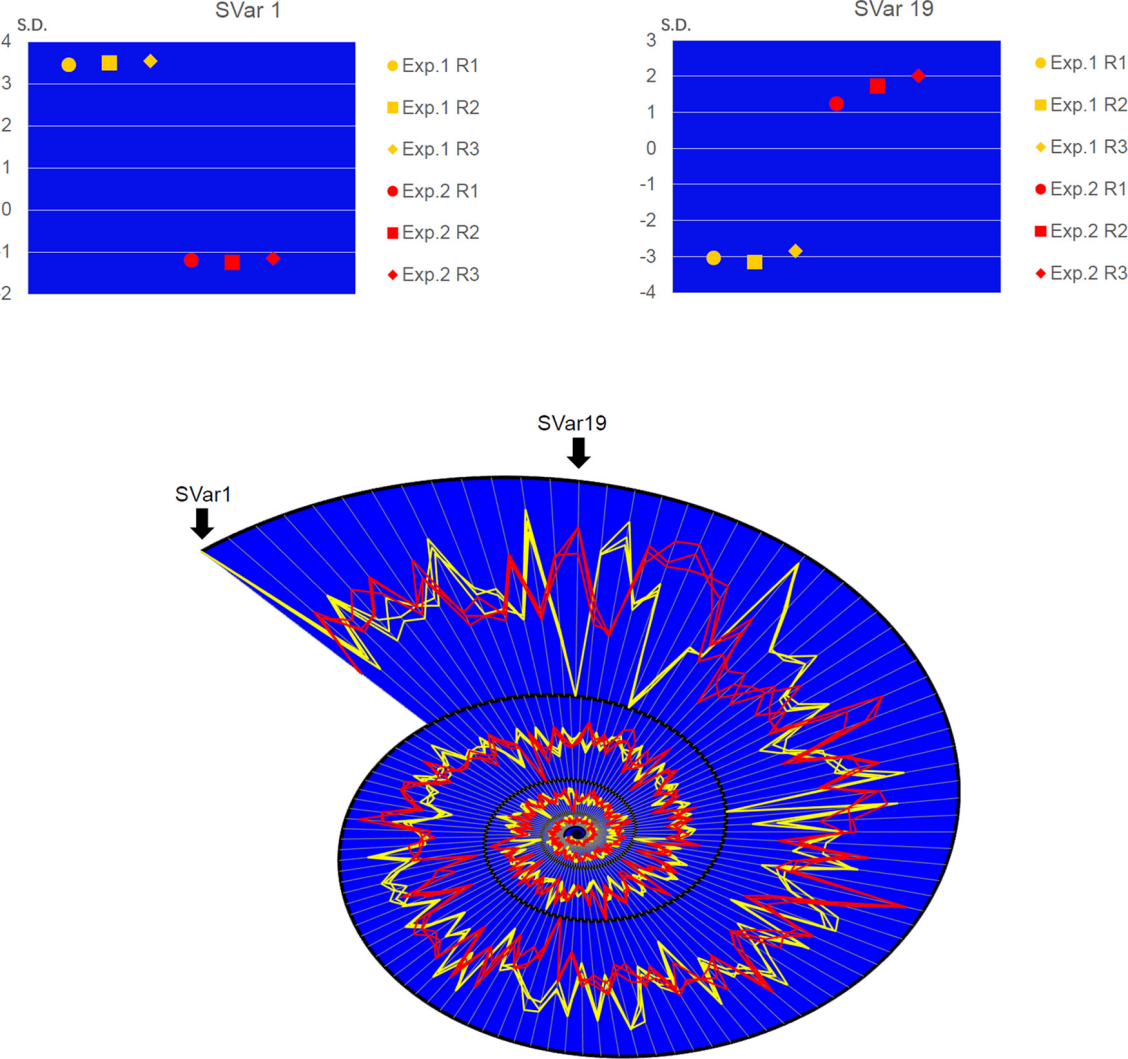
Parallel coordinate plot presenting normalized differences in gene expression for 3 replications (R1 to R3) of 2 experiments (Exp. 1 and 2) in a spiral form. Replications of experiment 1 are presented in yellow, while replications of experiment 2 are shown in red. In the 1st state variable, there was a significant difference between the 2 experiments. Furthermore, replications of experiment 2 showed more differences in the 19th state variable than those of experiment 1. The two small plots above show more details of the differences of standard deviation (S.D.) for the 1st and 19th state variables.

We investigated the transcriptional states for 3 replications of 2 published experiments (26). In Fig. 6, the parallel coordinate plot in a spiral form shows significant expression differences using TCSY. In the 1st dimension, there was a significant difference between the 2 experiments. In addition to the difference between experiments, the plot shows small expression differences among different replications, while expression was highly similar among the replications (Table 1). For example, replications of experiment 2 showed more differences in the 19th dimension (Fig. 6). Our results suggest that TCSY can present different transcriptional states and distinguish them from each other. To facilitate the application of TCSY, we developed an R package to map any transcriptional state of genome-wide expression data sets to the coordinate system (R package available at https://github.com/zyangx/ori).

**TABLE 1:** Pearson’s correlation coefficients for expression changes of 3 replications of 2 experiments

Experiment and replication	Pearson’s *r* for expression change in indicated experiment and replication^*a*^					
	Exp1.r1	Exp1.r2	Exp1.r3	Exp2.r1	Exp2.r2	Exp2.r3
Exp1.r1	1					
Exp1.r2	**0.981**	1				
Exp1.r3	**0.974**	**0.981**	1			
Exp2.r1	−0.682	−0.707	−0.702	1		
Exp2.r2	−0.683	−0.704	−0.694	**0.919**	1	
Exp2.r3	−0.663	−0.666	−0.661	**0.857**	**0.905**	1

a In experiment 1, changes in expression were obtained for wild-type yeasts before and after 20 min of stress treatment (0.4 M KCl); in experiment 2, a yeast strain lacking Hog1 was evaluated before and after the same stress treatment. Exp1 and -2, experiments 1 and 2; r1 to -3, replications 1 to 3. The boldface coefficients indicate the data correlations between the replications of the same experiments.

## DISCUSSION

Alter et al. (27) developed the “eigengene” and “eigenarray” to extract critical information from massive amounts of transcriptome data. Thereafter, investigators used singular value decomposition (SVD) and similar approaches to define different eigengenes in many studies (28–31). The different eigengenes are not comparable between different studies, however, because they are constructed separately using different experimental data. The lack of a universal underlying model is a significant limitation in gene expression data analysis. In the present study, we constructed a universal model using all of the published data available for S. cerevisiae and provide the model in an R package. The model can be applied to represent different data in the universal system TCSY (see Materials and Methods). In the coordinate system, transcriptional states can be compared in multiple dimensions between different data sets to disclose significant changes in gene expression. Each dimension of TCSY is associated with different GO terms and sequence motifs of different weights.

There is a close relationship between classic principal component analysis (PCA) and our approach using SVD to estimate the minimum size of state variables for the state-space representation of transcriptional regulation. It should be noted, however, that our results must be interpreted in a different manner than the classic PCA. The classic PCA is a model-free approach, and therefore, residual variance is usually explained by contributions of the rest of the principal components. Our study, however, is a model-based analysis where residual variance is due to system noise. That is, the residual variance of expression data in our model with 450 state variables cannot be explained by biological regulation. Therefore, our work should be interpreted cautiously according to the theory of classic PCA.

In studies of transcriptional regulation, information capacity measured in Shannon entropy is usually employed as a good measure of the diversity of gene expression. For example, Schug et al. (32) distinguished tissue-specific expression from ubiquitous genes in Shannon entropy. Furthermore, Martínez and Reyes-Valdés developed a framework to define diversity, specialization, and gene specificity in transcriptomes through information theory (33). More efforts were made to develop methods and tools with information theory to analyze all kinds of expression data (34–37). Previous studies, however supplied limited clues about the information capacity of sequence motifs. Our studies of the information capacity conceptualize the roles of the general motifs in transcriptional regulation.

Although the estimated size of state variables is much less than that of total genes in yeast, genetic regulation still properly responds to various environmental stimulations. Even if each state variable makes only a binary response to external signals, there are more than 2.91 × 10^125^ (2^450^) possible configurations for the 450 state variables. The large size of the configurations explains the limited information capacity of promoter motifs because gene transcription does not need to respond to each shift in the configuration. Reducing the information capacity of promoters could be an optimized evolutionary strategy to overcome the interference of regulatory noise. While multiple DNA binding factors play critical roles as members of the transcriptional complex, the poor information capacity of the corresponding motifs might not be a hindrance for proper regulation of mRNA transcription (38).

In this report, we show that the global gene expression in yeast can be effectively represented by no more than 450 state variables. The ability to represent different transcripts using the variables enables direct quantification of the differences between various transcriptional states. In a linear system, changes in gene expression can easily be normalized, scaled, and further compared with the state variables. Because the gene expression diversity is represented with only a few hundred variables, it is easy to visualize the differences between and within experiments in the universal system TCSY.

## MATERIALS AND METHODS

### DOF as the minimum number of state variables.

Gene expression changes over a discrete time period can be modeled by a state-space representation using the equations x(t + 1)=Ax(t) + Bu(t) and y(t)=Cx(t) + Du(t), where t is a moment in time, *y* is the observed gene expression level in logarithmic form, *x* is the vector of the state variables, and *u* is the inputs of control (experimental) parameters (39). *A* is the state matrix that represents the interaction between state variables, while *C* is the output matrix that determines the observed expression changes by the state variables. Both input matrix *B* and feedforward matrix *D* model how the inputs affect the state variables and observations. The feedforward matrix *D* could be 0 when feedforward is not present. Consequently, we can restate the state-space representation as follows:

(1) $$y(t)=C\sum_{i=0}^{t-1}A^{i}Bu(t-i)$$

When an experimental sample is treated (marked by * below) at an early moment in time *j* in a well-controlled study, the difference in the gene expression between experimental and control groups at time *t* is as follows:

(2) $$\Delta y=y^{*}(t)-y(t)=L_{A,B,C}(u^{*}-u)$$

where $L_{A,B,C}(\cdot )$ is a linear function with the combined information of matrices *A*, *B*, and *C* (equation 1). Without detailed knowledge about matrices *A*, *B*, and *C* and vector *x*, we can determine the DOF for transcriptional regulation, i.e., the minimum number of independent inputs μ and state variables *x* in the state-space representation. The DOF reflects the complexity of genetic regulation.

We transformed the differences in gene expression equation 2 to Δy=UΣV by singular value decomposition (SVD) (40). The vectors in *U* are called the left singular vectors, which represent the contribution of state variables (explanatory variables) in the linear function $L_{A,B,C}(\cdot )$, and the vectors in *V* are the right singular vectors, which represent various inputs from different experiments. The plots in Fig. 1 show that the state variables make different contributions to the expression of different genes. The diagonal entries of matrix Σ are the singular values of Δy. The number of significant eigenvalues is the suggested number of state variables in the model of gene expression (equation 2), i.e., the DOF of gene expression.

### Data.

We initially collected 11,483 gene expression data sets for S. cerevisiae from the Saccharomyces Genome Database (SGD) (41). A total of 6,692 genes were identified among all of the data sets, while some genes were not found in particular data sets. We removed the genes with a high missing rate (missing in more than 250 experiments) and experiments with insufficient gene data (<4,000 genes). To eliminate potential bias regarding data normalization, we further removed experimental data with a mean absolute logarithm expression difference larger than 0.5. As a result, 6,322 gene expression data sets with 4,529 genes were included for further analysis.

Genome sequences of S. cerevisiae (S288C, version R64) were also downloaded from the SGD website. The annotation for the SGD genes was obtained from the FTP site of GENOME golden path at the University of California, Santa Cruz (42). The ontology of the SGD genes was obtained from Gene Ontology Consortium (http://geneontology.org/docs/download-ontology/).

### Estimating the DOF of gene expression.

We determined the number of significant state variables by applying a search strategy with cross-validations. The cross-validation method identifies the size of state variables that best describe the systematic variations in data. It is commonly used in model-based studies, while model-free studies usually employ a variance threshold approach to determine the size of significant dimensions. A detailed introduction to the cross-validation method can be found in the work of Bro et al. (20).

For expression data matrix *X*, we divided the full data set into a training data set and a testing data set. The 2 data sets contained expression data of the same genes but from different experiments. The following procedure was applied to centralized data sets: (i) obtain right singular vectors *V* of the training data by SVD (Δy=UΣV); (ii) approximate the error E for a given number of components *k* in

$$E_{\text{approx}}=\sum_{i=1}^{n}{\left\|[I-PP^{T}+\text{diag}(PP^{T})]X_{\text{testing}}\right\|}^{2}$$

where *P* is size *k* for the most significant vectors (20); (iii) apply a golden section search algorithm to search for a proper *k* to minimize the errors (43); and (iv) repeat the above search process 15 times on different training and testing data sets to evaluate the robustness of the model across data sets and convergence of the search algorithm.

### Mapping genetic features to the model inputs.

The above-described linear model allowed us to map gene expression or other genetic features (such as sequence motifs and ontological terms) to the state variables of a model. When vector Δy is known to exist for each feature or genome-wide expression difference, vector $\hat{V}$, which presents independent state variables of the expression model, can be obtained by least-squares approximation in $\hat{V}={\left(X^{T}X\right)}^{-1}X^{T}\Delta y$, where matrix $X=U^{\left(m\right)}\Sigma^{\left(m\right)}$ is the basis of a universal coordinate system with *m* dimensions defined by the model. Because the number of genes involved in Δy is much higher than the number of significant state variables, the least-squares estimation of $\hat{V}$ is robust to missing elements in Δy.

### Calculating the information capacity of motifs for transcriptional regulation.

Each state variable was represented by a vector having 65,536 elements, each of which corresponds to 1 of the 4^8^ motifs. Elements whose corresponding motifs were ranked in the top 1% of positive-weight motifs were selected and given a value of 1, elements whose corresponding motifs were ranked in the top 1% of negative-weight motifs were selected and given a value of −1, and all remaining elements were given a value of 0. Consequently, we represented the 450 state variables as a 65,536-by-450 matrix. Rows of the matrix were categorized into different groups in which each group member had the same configuration of element values. The information capacity H of the selected motifs in the regulation system was obtained in the following equation:

$$H=-\sum_{i}p_{i}\log_{2}(p_{i})$$

where $p_{i}$ is the frequency of motif group *i* in the system. For the promoter of gene *j* with a total of *k* selected motifs, the information capacity of the presence of motifs was defined as

$$H(j)=-\frac{1}{k}\sum_{i=1}^{k}q{\left(j\right)}_{i}\log_{2}[q{\left(j\right)}_{i}]$$

where $q{\left(j\right)}_{i}$ is the probability that motif *i* of gene *j* appears in the selected motifs of the 450 state variables.

### Enrichment analysis of ranked gene list.

All involved genes were ranked by the aforementioned information capacities of their promoters. The enrichment analysis was applied to the ranked list using both GSEA (version 4.1.0) and g:Profiler (23, 44). Visualization of the enrichment data was conducted in Cytoscape (version 3.8.2) with the Enrichment Map plugin (24, 45).

### Statistical analysis.

The general statistical analysis and figure preparation for the manuscript were conducted in the R (version 3.5.2) and MATLAB (version 2018a) programming environments.

### Data availability.

Detailed information on the expression data used in this study is supplied in Table S1 in the supplemental material. All the data files are publicly available in the *Saccharomyces* Genome Database (https://www.yeastgenome.org/). All data and other material used in this study are publicly available upon request. The R package ORI is publicly available online at https://github.com/zyangx/ori. The package supplies functions to represent yeast expression data in TCSY (transcriptional coordinate system of yeast) as a parallel coordinate plot or to visualize the contribution of GO terms to each of the state variables of TCSY in a word cloud. All codes and other material used in this study are publicly available upon request.
