# Supplementary material for: Degree of Freedom of Gene Expression in Saccharomyces cerevisiae
Source: Microbiol Spectr. 2022 Mar 1;10(2):e00838-21. doi: 10.1128/spectrum.00838-21 (PMC9045123; doi:10.1128/spectrum.00838-21)
Supplement: SUPPLEMENTAL FILE 1 — Supplemental material. Download SPECTRUM00838-21_Supp_1_seq10.pdf, PDF file, 0.4 MB [file spectrum00838-21_supp_1_seq10.pdf]

Supplementary Materials for

Degree of Freedom of Gene Expression in *Saccharomyces cerevisiae*

Zhen Yang, Feng Xu, Aijuan Xue, Hong lv, Yungang He*

*Corresponding author. Email:heyungang@fudan.edu.cn

This PDF file includes:

Figure S1

Table S1

Figure S1. State variables of the model represented the transcriptional variances well. All the state variables are ranked on the x-axis. The variances explained by each variable are presented in blue on the left y-axis; the cumulative variance is shown in red on the right y-axis.

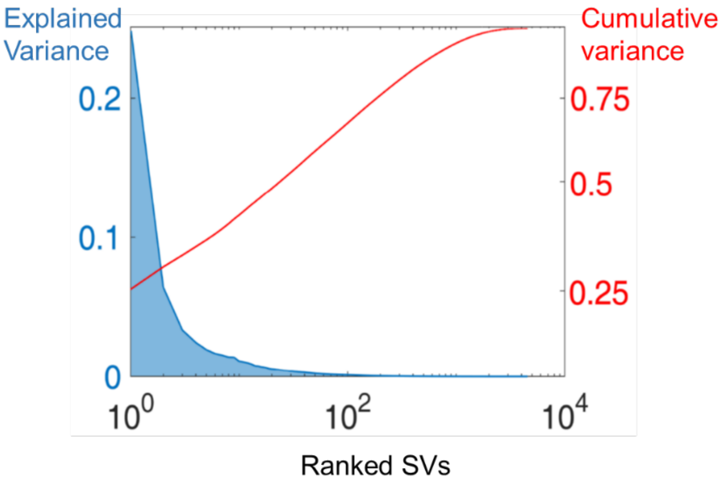

Table S1. Detailed information of the included data in the Saccharomyces Genome Database.

Publication Information	Name of data file in SGD	Number of experiments
**Abbott_2008_PMID_18676708**	GSE10066_setA_family.pcl	12
**Agarwal_2008_PMID_18156292**	GSE10073_setA_family.pcl	6
**Aguilera_2005_PMID_15780657**	GSE8900_setA_family.pcl	18
**Albulescu_2012_PMID_22479188**	GSE34330GPL8154.sfp.pcl	12
**Alff-Tuomala_2016_PMID_26454869**	GSE69966.mapped.pcl	30
**Almario_2013_PMID_23613173**	GSE44085.remapped.final.pcl	24
**Alper_2006_PMID_17158319**	GSE5185_setA_family.pcl	12
**Ambroset_2011_PMID_22384338**	GSE26437_final.pcl	40
**Anderson_2006_PMID_16896209**	GSE4261_setA_family.pcl	26
**Anderson_2009_PMID_19273689**	GSE12055_set0_family.pcl	46
**Angell_2006_PMID_17185230**	GSE6185_setA_family.pcl	6
**Angus-Hill_2001_PMID_11336698**	2010.Angus01.flt.knn.avg.pcl	8
**Ansari_2012_PMID_21971086**	GSE31774_final.pcl	21
**Apweiler_2012_PMID_22697265**	GSE33098_final.pcl	18
**Aragon_2006_PMID_16507144**	GSE3729_set6_family.pcl	78
**Aragon_2008_PMID_18199684**	GSE8624_set2_family.pcl	368
**Aristizabal_2013_PMID_24009531**	GSE43120.remapped.final.pcl	24
**Auld_2006_PMID_16543154**	GSE4272_set0_family.pcl	17
**Azzouz_2009_PMID_19155328**	GSE10521_setA_family.pcl	25
**Backhus_2001_PMID_12702356**	GSE4887_setA_family.pcl	6
**Baerends_2008_PMID_18378663**	GSE8902_setA_family.pcl	8
**Baetz_2001_PMID_11533240**	2010.Baetz01.flt.knn.avg.pcl	5
**Barbara_2007_PMID_17124610**	GSE5027_setA_family.pcl	12
**Barbosa_2015_PMID_25884705**	GSE63187.final.pcl	25
**Barbosa_2015_PMID_26314747**	GSE66521.mapped.pcl	16
**Barreto_2012_PMID_23039231**	GSE24712_final.pcl	18
**Batista-Nascimento_2013_PMID_23295455**	GSE33427.remapped.final.pcl	15

**Bedalov_2001_PMID_11752457**	2010.Bedalov01.flt.knn.avg.pcl	7
**Belli_2004_PMID_14722110**	2010.Belli04.filter.flt.knn.avg.div.log.pcl	9
**Benton_2006_PMID_17140446**	GSE6018_set1_family.pcl	13
**Bernstein_2000_PMID_11095743**	2010.Bernstein00_TSA.filter.flt.knn.avg.div.log.pcl	5
**Berry_2008_PMID_18753408**	GSE8335_set13_family.pcl	5
**Bessonov_2013_PMID_24130853**	GSE34117.remapped.final.pcl	14
**Bester_2012_PMID_22384390**	GSE29371_final.pcl	12
**Boer_2005_PMID_15949974**	2010.Boer05.filter.flt.knn.avg.div.log.pcl	12
**Boer_2007_PMID_17419774**	GSE6405_setA_family.pcl	15
**Borde_2009_PMID_19078966**	GSE10948_setA_family.pcl	7
**Braberg_2013_PMID_23932120**	GSE47429.remapped.final.pcl	57
**Bradley_2009_PMID_19180179**	GSE11754_set1_family.pcl	6
**Brauer_2005_PMID_15758028**	2010.Brauer05_batch2.flt.knn.avg.pcl	7
**Brauer_2008_PMID_17959824**	GSE8825_set5_family.pcl	6
**Braun_2013_PMID_23207903**	GSE40116_final.pcl	12
**Brem_2005_PMID_15659551**	2010.Brem05_orig.flt.knn.avg.pcl	131
**Brion_2013_PMID_24094006**	GSE41025.remapped.final.pcl	56
**Bristow_2014_PMID_25200947**	GSE49650.remapped.final.pcl	160
**Brown_2008_PMID_18422925**	GSE9875_setA_family.pcl	6
**Bro_2003_PMID_12791685**	2010.Bro03.filter.flt.knn.avg.div.log.pcl	7
**Bulik_2003_PMID_14555471**	2010.Bulik03.filter.flt.knn.avg.div.log.pcl	11
**Caba_2005_PMID_15878181**	2010.Caba05.filter.flt.knn.avg.div.log.pcl	24
**Canadell_2015_PMID_25425491**	GSE58985.final.pcl	3
**Capaldi_2008_PMID_18931682**	GSE12270_setA_family.pcl	85
**Carmel-Harel_2001_PMID_11169101**	2010.CarmelHarel01.flt.knn.avg.pcl	15
**Carroll_2001_PMID_11675494**	2010.Carroll01.log.flt.knn.avg.pcl	12
**Carter_2007_PMID_17389876**	GSE5938_setA_family.pcl	90
**Carter_2012_PMID_23071457**	GSE34787_final.pcl	96
**Casado_2011_PMID_21749328**	GSE27925_final.pcl	16
**Casamayor_2012_PMID_22372618**	GSE25697_final.pcl	12

**Causton_2001_PMID_11179418**	2010.Causton01_sorbitol.filter.flt.knn.avg.div.log.pcl	6
**Celona_2011_PMID_21738444**	GSE23711_final.pcl	6
**Chang_2013_PMID_23660188**	GSE45692.remapped.final.pcl	1
**Chang_2013_PMID_24051094**	GSE49340.remapped.final.pcl	1
**Chan_2008_PMID_18708563**	GSE12004_set1_family.pcl	5
**Chasman_2014_PMID_25411400**	GSE60700.final.pcl	9
**Chechik_2008_PMID_18953355**	GSE13219_set12_family.pcl	6
**Chen_2006_PMID_16397762**	GSE3160_setA_family.pcl	12
**Cheung_2008_PMID_18998772**	GSE12272_setA_family.pcl	6
**Chitikila_2002_PMID_12419230**	2010.Chitikila02.flt.knn.avg.pcl	20
**Chiu_2011_PMID_21147769**	GSE18334_final.pcl	18
**Cho_1998_PMID_9702192**	2010.Cho98.filter.flt.knn.avg.div.log.pcl	17
**Chua_2006_PMID_16880382**	GSE5499_set1_family.pcl	102
**Chujo_2015_PMID_25304510**	GSE55755.final.pcl	4
**Chumnanpuen_2012_PMID_22622761**	GSE32418_final.pcl	24
**Chu_1998_PMID_9784122**	2010.Chu98.flt.knn.avg.pcl	7
**Cipollina_2008_PMID_18174152**	GSE5238_setA_family.pcl	12
**Cipollina_2008_PMID_18524923**	GSE9644_set1_family.pcl	13
**Clark_2002_PMID_11988574**	2010.Clark02_orig.flt.knn.avg.pcl	17
**Cohen_2002_PMID_12006656**	2010.Cohen02.filter.flt.knn.avg.div.log.pcl	11
**Crosas_2015_PMID_25715111**	GSE45297.final.pcl	12
**Cullen_2004_PMID_15256499**	2010.glycosylation.pcl	2
**Daran-Lapujade_2004_PMID_14630934**	GSE8895_setA_family.pcl	12
**Daran-Lapujade_2007_PMID_17898166**	GSE9232_setA_family.pcl	9
**DeRisi_1997_PMID_9381177**	2010.diauxic.pcl	7
**Dettman_2007_PMID_17538619**	GSE6870_setA_family.pcl	8
**De_Nadal_2004_PMID_14737171**	2010.deNadal04.flt.knn.avg.pcl	12
**De_Nicola_2007_PMID_17933919**	GSE8089_setA_family.pcl	6
**Dion_2005_PMID_15795371**	GSE2324_setA_family.pcl	55
**Dominissini_2012_PMID_22575960**	GSE36958_final.pcl	9

**Dong_2007_PMID_17317628**	GSE6647_setA_family.pcl	10
**Dorfel_2016_PMID_27668839**	GSE86358.mapped.pcl	16
**Dori-Bachash_2011_PMID_21811398**	GSE29960_final.pcl	18
**Drozdova_2014_PMID_25227157**	GSE52189.remapped.final.pcl	12
**Dubacq_2006_PMID_16328372**	GSE1915_setA_family.pcl	16
**Duvel_2003_PMID_12820961**	2010.Duvel03_immediateRap.flt.knn.avg.pcl	10
**Dymond_2011_PMID_21918511**	GSE31326_final.pcl	15
**Epstein_2001_PMID_11179416**	2010.Epstein00.flt.knn.avg.pcl	11
**Eriksson_2005_PMID_16199888**	2010.Eriksson05.filter.flt.knn.avg.div.log.pcl	6
**Erlich_2008_PMID_19005567**	GSE12150_setA_family.pcl	12
**Felipe-Abrio_2015_PMID_25452497**	GSE55223.final.pcl	15
**Ferea_1999_PMID_10449761**	2010.evolution.pcl	4
**Fleming_2002_PMID_11830665**	2010.Fleming99.log.flt.knn.avg.pcl	30
**Flom_2012_PMID_22461145**	GSE34757_final.pcl	6
**Friedlander_2006_PMID_16542486**	GSE3820_setA_family.pcl	27
**Fry_2003_PMID_12875747**	2010.Fry03.filter.flt.knn.avg.div.log.pcl	8
**Fry_2006_PMID_17163986**	GSE5301_setA_family.pcl	18
**Gaillard_2014_PMID_24603480**	GSE50947.remapped.final.pcl	11
**Galitski_1999_PMID_10398601**	2010.ploidy.pcl	11
**Garcia-Martinez_2016_PMID_26717982**	GSE65225.mapped.pcl	6
**Garcia-Martinez_2016_PMID_27648972**	GSE72356.mapped.pcl	20
**Garcia-Oliver_2013_PMID_23599000**	GSE39861.remapped.final.pcl	32
**Garcia-Rodriguez_2012_PMID_22493290**	GSE29420_final.pcl	6
**Garcia_2009_PMID_19234305**	GSE12684_setA_family.pcl	18
**Gardner_2005_PMID_15797381**	GSE2159_setA_family.pcl	12
**Gardner_2005_PMID_15988024**	GSE2330_setA_family.pcl	24
**Garrido-Godino_2016_PMID_27001033**	GSE65283.mapped.pcl	18
**Gasch_2000_PMID_11102521**	2010.Gasch00_steadyState(y14).flt.knn.avg.pcl	8
**Gasch_2001_PMID_11598186**	2010.DNAdamage.pcl	52
**Gavalda_2016_PMID_27035147**	GSE68487.mapped.pcl	6

**Geijer_2012_PMID_23066959**	GSE29960_final.pcl	18
**Ghazal_2005_PMID_15798187**	GSE65111.final.pcl	8
**Gil_2011_PMID_21842488**	GSE28677_final.pcl	12
**Gitter_2013_PMID_23064748**	GSE28213GPL13340.sfp.pcl	16
**Golla_2015_PMID_25919230**	GSE62399.final.pcl	4
**Golla_2016_PMID_27734932**	GSE62400.mapped.pcl	4
**Gomar-Alba_2015_PMID_26048894**	GSE59659.final.pcl	4
**Gomez-Gonzalez_2011_PMID_21701562**	GSE24802_final.pcl	10
**Gonzalez-Aguilera_2011_PMID_21680710**	GSE26303_final.pcl	6
**Gonzalez_2006_PMID_16973600**	GSE6801_setA_family.pcl	10
**Gonzalez_2013_PMID_23704987**	GSE38260.remapped.final.pcl	14
**Goossens_2015_PMID_25873380**	GSE64468.final.pcl	17
**Gresham_2008_PMID_19079573**	GSE13435_set0_family.pcl	63
**Gross_2000_PMID_10922376**	2010.Gross00.flt.knn.avg.pcl	6
**Grund_2008_PMID_18762579**	GSE6370_set0_family.pcl	6
**Guan_2006_PMID_17166056**	GSE3076_setA_family.pcl	96
**Guan_2012_PMID_22851651**	GSE32196GPL15778.sfp.pcl	11
**Guo_2006_PMID_16122766**	GSE2420_setA_family.pcl	11
**Hao_2012_PMID_22179789**	GSE32703_final.pcl	56
**Hardwick_1999_PMID_10611304**	2010.Hardwick00.flt.knn.avg.pcl	14
**Hasunuma_2014_PMID_23916856**	GSE45273.remapped.final.pcl	12
**Haugen_2004_PMID_15575969**	2010.arsenic_time_dose.pcl	4
**Hazelwood_2008_PMID_18281432**	GSE9590_setA_family.pcl	12
**Hebly_2014_PMID_24814792**	GSE55372.remapped.final.pcl	17
**Herrera-Moyano_2014_PMID_24636987**	GSE54340.remapped.final.pcl	9
**Hickman_2007_PMID_17785431**	GSE8613_setA_family.pcl	6
**Hodges_2015_PMID_25971662**	GSE68159.final.pcl	6
**Hoffman_2015_PMID_25609572**	GSE64446.final.pcl	2
**Holbein_2009_PMID_19324962**	GSE14619_setA_family.pcl	9
**Hong_2011_PMID_21715660**	GSE27185_final.pcl	12

**Hong_2012_PMID_22904057**	GSE36118_final.pcl	14
**Hou_2014_PMID_24237754**	GSE39311.remapped.final.pcl	12
**Huang_2002_PMID_12077337**	2010.Huang02.flt.knn.avg.pcl	20
**Huang_2004_PMID_15539461**	2010.rapamycin.pcl	10
**Hughes_2000_PMID_10929718**	2010.Hughes00.flt.knn.avg.pcl	300
**Huisinga_2007_PMID_17407552**	GSE7385_setA_family.pcl	149
**Hu_2007_PMID_17417638**	GSE4654_setA_family.pcl	269
**Ideker_2001_PMID_11340206**	2010.Ideker01.flt.knn.avg.pcl	21
**Irvin_2006_PMID_16407318**	GSE3006_setA_family.pcl	14
**Iwahashi_2007_PMID_17408496**	GSE6118_setA_family.pcl	15
**Iwahashi_2007_PMID_18075112**	GSE8718_setA_family.pcl	9
**Jaehnig_2013_PMID_23810556**	GSE40351.remapped.final.pcl	72
**James_2007_PMID_17660549**	GSE9432_setA_family.pcl	8
**Jansen_2004_PMID_15066785**	GSE8897_setA_family.pcl	5
**Jansen_2005_PMID_15870473**	GSE8898_setA_family.pcl	6
**Jimeno_2011_PMID_21149575**	GSE18994_final.pcl	6
**Jin_2004_PMID_15528549**	2010.Jin04.filter.flt.knn.avg.div.log.pcl	6
**Jin_2007_PMID_17664279**	GSE6331_setA_family.pcl	20
**Johanson_2006_PMID_16440349**	GSE2818_set0_family.pcl	6
**Johansson_2007_PMID_18087042**	GSE9482_setA_family.pcl	40
**Jones_2003_PMID_14570984**	2010.Jones03_orig.flt.knn.avg.pcl	5
**Joseph-Strauss_2007_PMID_17999778**	GSE7393_set5_family.pcl	7
**Kao_2008_PMID_19029899**	GSE11071_setA_family.pcl	29
**Kaplan_2008_PMID_19023413**	GSE12822_setA_family.pcl	34
**Keller_2001_PMID_11504737**	2010.Keller01.flt.knn.avg.pcl	4
**Kelly_2006_PMID_16720269**	GSE4311_setA_family.pcl	6
**Khakhina_2015_PMID_25724885**	GSE65264.final.pcl	8
**Kim_2009_PMID_19456872**	GSE14102_setA_family.pcl	9
**Kitagawa_2002_PMID_12269742**	GSE9336_setA_family.pcl	9
**Kitagawa_2003_PMID_12854720**	GSE9337_setA_family.pcl	15

**Kitagawa_2005_PMID_16377885**	GSE9401_setA_family.pcl	27
**Klecker_2015_PMID_26669658**	GSE49580.mapped.pcl	24
**Klevecz_2004_PMID_14734811**	GSE2583_setA_family.pcl	32
**Klockow_2008_PMID_18455824**	GSE6358_setA_family.pcl	7
**Knijnenburg_2007_PMID_17241460**	GSE4807_setA_family.pcl	30
**Knijnenburg_2009_PMID_19173729**	GSE11452_setA_family.pcl	170
**Komili_2007_PMID_17981122**	GSE8761_setA_family.pcl	24
**Kresnowati_2006_PMID_16969341**	GSE3821_setA_family.pcl	16
**Kugler_2016_PMID_27245696**	GSE75462.mapped.pcl	23
**Kugou_2007_PMID_17396017**	GSE6620_setA_family.pcl	8
**Kuhn_2001_PMID_11154278**	2010.Kuhn01.flt.knn.avg.pcl	3
**Kundaje_2008_PMID_19008939**	GSE8343_setA_family.pcl	24
**Kuranda_2006_PMID_16925551**	GSE4049_setA_family.pcl	36
**Kvitek_2008_PMID_18927628**	GSE10269_set0_family.pcl	9
**Lai_2006_PMID_16963631**	2010.glucose_anoxia_reoxygenation.pcl	24
**Lai_2008_PMID_19105839**	GSE3706_setA_family.pcl	227
**Lai_2013_PMID_23893744**	GSE48956.remapped.final.pcl	4
**Landry_2006_PMID_16427747**	GSE3021_setA_family.pcl	91
**Landry_2007_PMID_17525304**	GSE7537_setA_family.pcl	10
**Landry_2014_PMID_24714560**	GSE55121.remapped.final.pcl	32
**Lanza_2012_PMID_22558379**	GSE26923_final.pcl	15
**Larsson_2013_PMID_23991176**	GSE47712.remapped.final.pcl	12
**Leber_2004_PMID_15314654**	2010.Leber04.flt.knn.avg.pcl	13
**Lee_2000_PMID_12760044**	2010.HO_endonuclease.pcl	8
**Lee_2005_PMID_15989963**	2010.Lee05.filter.flt.knn.avg.div.log.pcl	9
**Lenstra_2011_PMID_21596317**	GSE25909_s313_final.pcl	313
**Lenstra_2013_PMID_24324601**	GSE40254.remapped.final.pcl	22
**Levy_2007_PMID_17327914**	GSE6302_set10_family.pcl	8
**Lewis_2014_PMID_24970865**	GSE54196.remapped.final.pcl	196
**Liang_2013_PMID_23985319**	GSE39950.remapped.final.pcl	6

**Licursi_2014_PMID_24164706**	GSE51563.remapped.final.pcl	2
**Liko_2007_PMID_17616518**	GSE8379_setA_family.pcl	8
**Ling_2013_PMID_23826995**	GSE38653.final.pcl	8
**Lin_2012_PMID_22308403**	GSE31390_final.pcl	6
**Liu_2013_PMID_23435897**	GSE38848.remapped.final.pcl	18
**Li_2006_PMID_17043222**	GSE9302_setA_family.pcl	48
**Lopez-Malo_2015_PMID_26194190**	GSE67428.final.pcl	6
**Lopez_2000_PMID_10940042**	2010.Lopez00.filter.flt.knn.avg.div.log.pcl	17
**Lu_2014_PMID_24631914**	GSE54741.remapped.final.pcl	27
**Lu_2016_PMID_27812096**	GSE76597.mapped.pcl	63
**Lyons_2000_PMID_10884426**	2010.Lyons00.flt.knn.avg.pcl	9
**Macadangdang_2014_PMID_24939988**	GSE50440.remapped.final.pcl	11
**Madhani_1999_PMID_10535956**	2010.Madhani99.filter.flt.knn.avg.div.log.pcl	11
**Malagon_2006_PMID_16510790**	GSE2831_setA_family.pcl	14
**Marks_2008_PMID_18215224**	GSE8536_setA_family.pcl	21
**Marsit_2016_PMID_26549518**	GSE62446.mapped.pcl	15
**Martin_2004_PMID_15476558**	2010.Martin04.filter.flt.knn.avg.div.log.pcl	12
**Marton_1998_PMID_9809554**	2010.Marton98.log.flt.knn.avg.pcl	7
**Matsumoto_2005_PMID_16209719**	GSE3316_setA_family.pcl	12
**Medintz_2007_PMID_17700863**	GSE7820_setA_family.pcl	12
**Menacho-Marquez_2007_PMID_17890903**	GSE8111_setA_family.pcl	4
**Mendes-Ferreira_2007_PMID_17601813**	GSE5842_set0_family.pcl	33
**Mendes_2013_PMID_23872557**	GSE45776.remapped.final.pcl	6
**Mendiratta_2006_PMID_16415340**	GSE3354_setA_family.pcl	9
**Meneghini_2003_PMID_12628191**	2010.swr1_htz1_hmr_sir2.pcl	24
**Miller-Fleming_2014_PMID_24706893**	GSE56124.remapped.final.pcl	12
**Mith_2015_PMID_25755096**	GSE63395.final.pcl	8
**Miyake_2004_PMID_15192094**	2010.Miyake04.flt.knn.avg.pcl	4
**Mizuguchi_2004_PMID_14645854**	2010.swr1_htz1_ino80.pcl	3
**Mnaimneh_2004_PMID_15242642**	2010.EssentialGenes.pcl	215

**Mojzita_2014_PMID_25192596**	GSE52736.final.pcl	36
**Molina-Navarro_2008_PMID_18424442**	GSE9663_set0_family.pcl	18
**Moreno-Cermeno_2013_PMID_24100161**	GSE44871.remapped.final.pcl	6
**Morillo-Huesca_2010_PMID_20711347**	GSE21571_final.pcl	24
**Munding_2010_PMID_21123654**	GSE24681_final.pcl	14
**Mutiu_2007_PMID_17447102**	GSE6316_setA_family.pcl	9
**Mutiu_2007_PMID_17660562**	GSE6847_setA_family.pcl	6
**Nag_2008_PMID_18508805**	GSE11282_setA_family.pcl	6
**Navarro-Tapia_2016_PMID_26925053**	GSE44863.mapped.pcl	8
**Navlakha_2012_PMID_22916002**	GSE28213GPL14666.sfp.pcl	12
**Ng_2013_PMID_24037263**	GSE39903.remapped.final.pcl	22
**Niles_2012_PMID_22307609**	GSE33185_final.pcl	9
**Noble_2015_PMID_25947166**	GSE55083.final.pcl	2
**O'Doherty_2013_PMID_24074273**	GSE47820.remapped.final.pcl	6
**O'Duibhir_2014_PMID_24952590**	GSE54539.remapped.final.pcl	12
**O'Rourke_2004_PMID_14595107**	2010.ORourke03.flt.knn.avg.pcl	133
**Oberstrass_2006_PMID_16429156**	GSE3859_setA_family.pcl	5
**Oeser_2016_PMID_26800527**	GSE57476.mapped.pcl	23
**Ogawa_2000_PMID_11102525**	2010.Ogawa00_set2.flt.knn.avg.pcl	3
**Olesen_2002_PMID_12702272**	2010.Olesen02.filter.flt.knn.avg.div.log.pcl	12
**Oliveira_2015_PMID_25888284**	GSE54851.final.pcl	14
**Orlandi_2004_PMID_14623890**	2010.Orlandi04.filter.flt.knn.avg.div.log.pcl	4
**Orlando_2008_PMID_18463633**	GSE8799_setA_family.pcl	60
**Oromendia_2012_PMID_23222101**	GSE40073_final.pcl	20
**Pan_2008_PMID_18366703**	GSE10554_setA_family.pcl	6
**Papini_2010_PMID_20815084**	GSE22122_final.pcl	6
**Parra_2006_PMID_16648479**	GSE3806_setA_family.pcl	20
**Parra_2007_PMID_17724083**	GSE7338_setA_family.pcl	6
**Pastor-Flores_2015_PMID_26391581**	GSE32623.final.pcl	8
**Pelechano_2008_PMID_17914747**	GSE8629_setA_family.pcl	6

**Penheiter_2005_PMID_16246724**	GSE3200_set1_family.pcl	5
**Pitkanen_2004_PMID_15520001**	2010.Pitkanen04.filter.flt.knn.avg.div.log.pcl	15
**Pramila_2002_PMID_12464633**	GSE5283_setA_family.pcl	13
**Pramila_2006_PMID_16912276**	GSE4987_setA_family.pcl	50
**Primig_2000_PMID_11101837**	2010.Primig00.filter.flt.knn.avg.div.log.pcl	24
**Prinz_2004_PMID_14993204**	2010.Prinz04.flt.knn.avg.pcl	10
**Protchenko_2001_PMID_11673473**	2010.Protchenko01.flt.knn.avg.pcl	4
**Protchenko_2008_PMID_18326586**	GSE9514_setA_family.pcl	8
**Puig_2008_PMID_18522836**	GSE11236_setA_family.pcl	9
**Rachfall_2013_PMID_23071099**	GSE34286_final.pcl	12
**Reinke_2006_PMID_16923813**	GSE4586_setA_family.pcl	6
**Renaud-Young_2015_PMID_25701288**	GSE66176.final.pcl	16
**Reyes_2014_PMID_24262517**	GSE51613.remapped.final.pcl	13
**Rintala_2011_PMID_21348598**	GSE22832_final.pcl	28
**Robertson_2000_PMID_10811893**	2010.Robertson00.filter.flt.knn.avg.div.log.pcl	12
**Roberts_2000_PMID_10657304**	2010.alpha_time.pcl	7
**Roberts_2006_PMID_16741729**	2010.glucose_glycerol_transition.pcl	5
**Robinson_2015_PMID_26130709**	GSE65365.final.pcl	16
**Rodriguez-Lombardero_2014_PMID_24763424**	GSE41094.remapped.final.pcl	18
**Rodriguez-Navarro_2004_PMID_14718168**	2010.RodriguezNavarro04.filter.flt.knn.avg.div.log.pcl	6
**Rodriguez-Quinones_2009_PMID_19426543**	GSE5931_setA_family.pcl	6
**Rollero_2016_PMID_26861624**	GSE68354.mapped.pcl	10
**Romagnoli_2014_PMID_24733517**	GSE52256.remapped.final.pcl	4
**Ronen_2006_PMID_16381818**	GSE4158_set0_family.pcl	26
**Rosaleny_2005_PMID_16023114**	GSE2434_setA_family.pcl	15
**Rossmann_2011_PMID_21474074**	GSE27222_final.pcl	12
**Rossouw_2009_PMID_19711068**	GSE11651_setA_family.pcl	43
**Roy_2013_PMID_23640720**	GSE38478GPL9294.remapped.sfp.pcl	32
**Ro_2008_PMID_18983675**	GSE11620_setA_family.pcl	14
**Rudra_2005_PMID_15692568**	2010.Rudra05.filter.flt.knn.avg.div.log.pcl	6

**Sabet_2004_PMID_15456858**	2010.Sabet04.filter.flt.knn.avg.div.log.pcl	18
**Saint_2014_PMID_24550006**	GSE44544.remapped.final.pcl	14
**Saldanha_2004_PMID_15240820**	2010.Saldanha04_UraSulPhoLeuComp.flt.knn.avg.pcl	24
**Salusjarvi_2008_PMID_18533012**	GSE12890_setA_family.pcl	15
**Sameith_2015_PMID_26700642**	GSE42536.mapped.pcl	287
**Santos-Pereira_2013_PMID_24240235**	GSE50186.remapped.final.pcl	6
**Sanz_2012_PMID_22621902**	GSE31176_final.pcl	18
**Sapra_2004_PMID_15452114**	2010.Sapra04.flt.knn.avg.pcl	24
**Sariki_2016_PMID_27718307**	GSE75447.mapped.pcl	4
**Schawalder_2004_PMID_15616569**	2010.Schawalder04.filter.flt.knn.avg.div.log.pcl	24
**Scherens_2006_PMID_16879428**	GSE2891_setA_family.pcl	18
**Schosserer_2015_PMID_25635753**	GSE63030.final.pcl	24
**Schrader_2006_PMID_16987817**	GSE5290_setA_family.pcl	8
**Segal_2003_PMID_12740579**	2010.Segal03_stationaryPhaseYPL230W.flt.knn.avg.pcl	12
**Shakoury-Elizeh_2004_PMID_14668481**	2010.ShakouryElizeh04.flt.knn.avg.pcl	6
**Shalem_2008_PMID_18854817**	GSE12222_set5_family.pcl	7
**Shalem_2011_PMID_21931566**	GSE26829_final.pcl	58
**Shapira_2004_PMID_15371544**	2010.Shapira04.flt.knn.avg.pcl	70
**Sha_2013_PMID_24073228**	GSE7645.remapped.final.pcl	48
**Sheltzer_2012_PMID_22802626**	GSE35853_final.pcl	8
**Shirra_2008_PMID_18955495**	GSE12061_setA_family.pcl	4
**Shivaswamy_2008_PMID_18212068**	GSE7665_set0_family.pcl	19
**Simmons_Kovacs_2012_PMID_22306294**	GSE32974_final.pcl	48
**Simons_2006_PMID_16870766**	GSE4669_setA_family.pcl	12
**Singh_2005_PMID_16332871**	GSE1313_setA_family.pcl	24
**Slavov_2014_PMID_24767987**	GSE56773.remapped.final.pcl	8
**Smith_2002_PMID_12135984**	2010.peroxisome.pcl	8
**Smith_2007_PMID_17551510**	GSE5862_set0_family.pcl	20
**Smith_2008_PMID_18416601**	GSE9376_setA_family.pcl	246
**Spedale_2012_PMID_21976730**	GSE30148_final.pcl	12

**Spellman_1998_PMID_9843569**	2010.Spellman98_elutriation.flt.knn.avg.pcl	14
**Sprouse_2009_PMID_19098311**	GSE12371_setA_family.pcl	6
**Stern_2007_PMID_17453047**	GSE6450_setA_family.pcl	22
**Stovicek_2014_PMID_24533484**	GSE40625.remapped.final.pcl	18
**Sudarsanam_2000_PMID_10725359**	2010.snf_swi_mutants.pcl	12
**Suzuki_2012_PMID_22897823**	GSE36954.remapped.final.pcl	18
**Suzuki_2013_PMID_23325148**	GSE36955.remapped.final.pcl	6
**Suzuki_2015_PMID_25609182**	GSE63663.final.pcl	6
**Tai_2005_PMID_15496405**	2010.Tai05.filter.flt.knn.avg.div.log.pcl	24
**Takagi_2005_PMID_15837426**	2010.Takagi05.filter.flt.knn.avg.div.log.pcl	12
**Takahashi_2011_PMID_21474073**	GSE27234_final.pcl	8
**Tanaka_2006_PMID_16943074**	GSE3043_setA_family.pcl	6
**Tesniere_2013_PMID_23658613**	GSE42027.remapped.final.pcl	4
**Thibault_2011_PMID_22143797**	GSE33844_final.pcl	15
**Thibault_2012_PMID_23000174**	GSE39419_final.pcl	16
**Thierry_2015_PMID_25635677**	GSE64431.final.pcl	4
**Thorsen_2007_PMID_17327492**	GSE6129_set3_family.pcl	6
**Tirosh_2008_PMID_18197176**	GSE7525_set0_family.pcl	6
**Tompa_2007_PMID_17179083**	GSE4934_setA_family.pcl	8
**Torres_2007_PMID_17702937**	GSE7812_set1_family.pcl	18
**Travers_2000_PMID_10847680**	2010.unfolded_protein_response.pcl	10
**Travesa_2012_PMID_22333915**	GSE33695_final.pcl	61
**Tsang_2014_PMID_24647101**	GSE55081.remapped.final.pcl	4
**Tu_2005_PMID_16254148**	GSE3431_setA_family.pcl	36
**Tyo_2012_PMID_22380681**	GSE27062_final.pcl	18
**Urban_2007_PMID_17560372**	GSE7660_set2_family.pcl	6
**van_Attikum_2004_PMID_15607975**	2010.MMSresponse.pcl	3
**van_den_Brink_2008_PMID_18304306**	GSE8187_setA_family.pcl	13
**van_de_Pasch_2013_PMID_23785440**	GSE33929.remapped.final.pcl	34
**Vemuri_2007_PMID_17287356**	GSE6277_setA_family.pcl	6

**Venkatasubrahmanyam_2007_PMID_17925448**	GSE4826_setA_family.pcl	16
**Verzijlbergen_2011_PMID_21998594**	GSE30168_final.pcl	36
**Vizoso-Vazquez_2012_PMID_22189861**	GSE30046_final.pcl	12
**Vos_2015_PMID_26369953**	GSE65942.mapped.pcl	16
**Wang_2002_PMID_11972065**	GSE2986_setA_family.pcl	58
**Wenger_2011_PMID_21829391**	GSE25081_final.pcl	36
**Williams_2002_PMID_12370439**	2010.Williams02.filter.flt.knn.avg.div.log.pcl	8
**Willis_2008_PMID_18604275**	GSE11397_setA_family.pcl	24
**Worley_2013_PMID_23643537**	GSE45370.remapped.final.pcl	55
**Wu_2008_PMID_18673560**	GSE11983_setA_family.pcl	4
**Wyrick_1999_PMID_10586882**	2010.histone.pcl	7
**Xue-Franzen_2013_PMID_23865462**	GSE36599.remapped.final.pcl	10
**Yamamoto_2005_PMID_15647283**	2010.Yamamoto05.filter.flt.knn.avg.div.log.pcl	4
**Yamane-Sando_2014_PMID_24510621**	GSE42083.remapped.final.pcl	1
**Yarragudi_2007_PMID_17158163**	GSE6073_setA_family.pcl	12
**Yeang_2005_PMID_15998451**	2010.Yeang05.flt.knn.avg.pcl	7
**Yiu_2008_PMID_18245757**	GSE10018_set3_family.pcl	6
**Yona_2012_PMID_23197825**	GSE40817_final.pcl	40
**Yoshimoto_2002_PMID_12058033**	2010.Yoshimoto02_Na.flt.knn.avg.pcl	16
**Yun_2000_PMID_10744769**	2010.Yun00.flt.knn.avg.pcl	4
**Yu_2006_PMID_16461773**	GSE4135_setA_family.pcl	14
**Zeng_2016_PMID_27485516**	GSE80748.mapped.pcl	8
**Zhang_2011_PMID_22068328**	GSE24421_final.pcl	24
**Zhang_2015_PMID_25897127**	GSE50492.final.pcl	10
**Zhou_2011_PMID_21700227**	GSE23580_final.pcl	66
**Zhu_2000_PMID_10894548**	GSE3182_setA_family.pcl	26
**Zhu_2008_PMID_18552845**	GSE11111_setA_family.pcl	9
**Zhu_2009_PMID_19158363**	GSE13684_setA_family.pcl	24
**Zhu_2015_PMID_26138482**	GSE65666.final.pcl	6
